# Topiramate-induced angle closure with acute myopia, macular striae

**DOI:** 10.4103/0974-620X.60018

**Published:** 2010

**Authors:** S. Natesh, S. K. Rajashekhara, A. S. D. Rao, B. Shetty

**Affiliations:** Departments of Vitreoretina, Glaucoma and Cataract and Refractive Surgery Narayana Nethralaya, Bangalore, India

**Keywords:** Topiramate, macular striae, optical coherence tomography

## Abstract

Topiramate is a sulfamate-substituted monosaccharide used in the treatment of seizures, and prophylaxis of migraine. A number of ocular side-effects have been described with use of topiramate, like bilateral angle closure, acute myopia and macular striae. Ultrasound biomicroscopy (UBM) clinches the diagnosis after ruling out other causes of shallow anterior chamber. Previous studies have not demonstrated internal limiting membrane folds presenting as macular striae. We report a case of topiramate-induced acute myopia with angle closure and macular striae in a young adult. This is the first report wherein striae formation after low doses of topiramate and their resolution have been documented by Optical Coherence Tomography (OCT).

## Introduction

Topiramate is a sulfamate-substituted drug used primarily for the treatment of seizures and as prophylaxis for migraine headaches. The doses used commonly are in the range of 50 mg - 400 mg per day. A number of ocular side-effects have been described with use of topiramate, namely bilateral angle closure, acute myopia and macular striae.[1,2]

Drug-induced myopia has been described with the use of topiramate, acetazolamide, spiranolactone, corticosteroids, sulfamethoxazole/trimethoprim, indapamide, promethazine, spironolactone, isosorbide-dinitrate and bromocriptine, tetracycline, hydro-chlorothiazide, penicillamine, quinine, metronidazole, isotretinoin, and aspirin.[3,4] The basic mechanism underlying drug-induced acute myopia with topiramate seems to be ciliary effusion causing ante-version of the ciliary body and anterior displacement of the iris-lens diaphragm. This induces bilateral acute myopia, non pupillary block angle closure, and raised intraocular pressure (IOP).[5]

We report a case wherein low doses of topiramate caused angle closure with acute myopia, documented with ultrasound biomicroscopy (UBM). The drug also caused development of macular striae, which was noted clinically and documented with optical coherence tomography (OCT). This is the first OCT documented report of macular striae with use of topiramate and its resolution.

## Case Report

A 23-year-old male presented with blurred vision in both eyes (OU) of two days duration. This was accompanied by mild redness and discomfort OU. There was a previous documentation of his refractive error, which was less than one diopter of myopia OU. He was on oral topiramate, 25 mg once daily for vascular migraine since five days.

On examination, his vision showed improvement to 20/20 OU with myopic correction of –6 diopters OU. Anterior segment showed bilateral shallow angles and gonioscopy showed occludable angles opening up to Schwalbe′s line. There was no pupillary abnormality. His intraocular pressure (IOP) was 24 mm of Hg OU. Fundus evaluation showed attached retina, healthy optic discs and macular striae.

**Figure 1:**
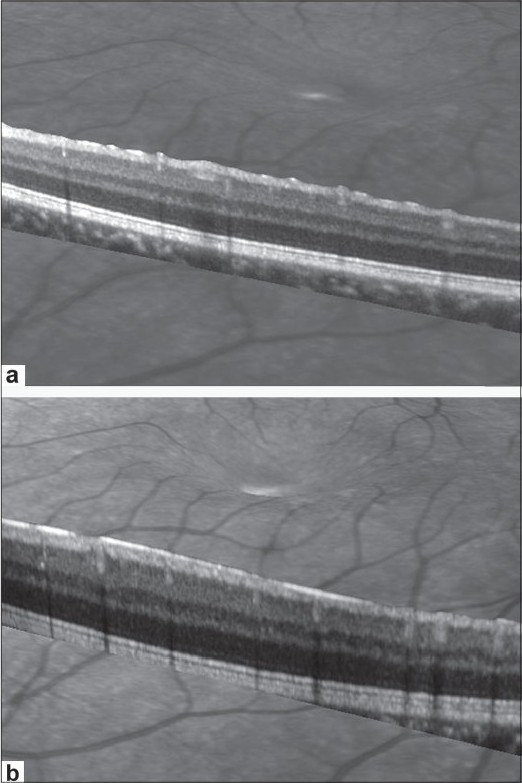
Scanning laser ophthalmoscope and optical coherence tomography images of the fundus OS showing (a) internal limiting membrane folds at the macula and (b) resolution of internal limiting membrane folds after cessation of topiramate

**Figure 2:**
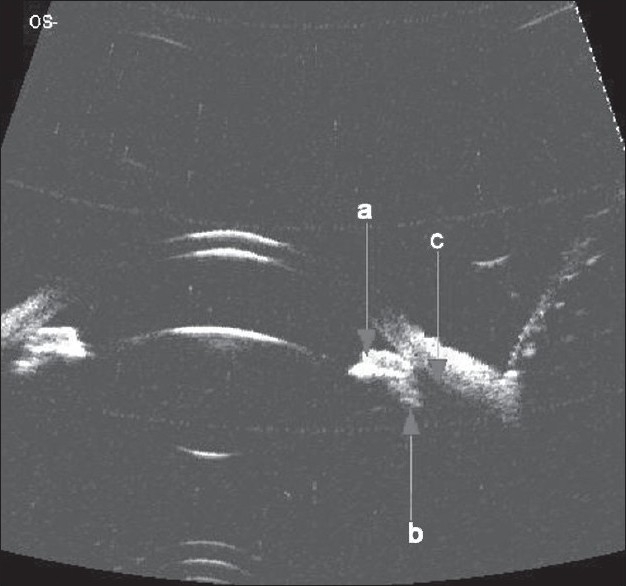
Ultrasound Biomicroscopy picture of the anterior segment OS showing the; (a) iris; (b) anteriorly rotated ciliary body and; (c) supraciliary effusion. Note the angle closure

Color vision was normal and optical coherence tomography (OCT) (spectralis HRA-OCT, Heidelberg engineering) showed striae at the level of the internal limiting membrane [Figure 1a]. No subfoveal fluid was noted. Ultra sound biomicroscopy (35 Hz, OTI, Canada) showed angle closure, anteversion of the ciliary body with ciliary effusion [Figure 2]. B scan ultrasonography showed diffuse choroidal effusion. The patient was asked to discontinue topiramate and was treated with topical cycloplegics and corticosteroid drops. IOP was 14 mm Hg OU next day and anterior chamber was better formed. In two days his vision recovered to 20/20. Macular striae resolved [Figure 1b] and gonioscopy showed open angles up to the scleral spur.

## Discussion

Angle closure and acute myopia occurs due to ciliary effusion, as an idiosyncratic reaction to topiramate.[3] Weak carbonic anhydrase activity of the topiramate and prostaglandin mediated effect is implicated to be the cause of ciliochoroidal effusion.[5,6] Prompt resolution following early discontinuation of the drug is the norm although some patients need IOP lowering medications. Peripheral iridectomy is of doubtful value. Ciliary effusion seen well on the UBM, clinches the diagnosis, ruling out other causes of shallow anterior chamber such as accommodative spasm, and primary angle closure.

Topiramate is commonly used in the range of 50 - 100 mg per day although some neurologists prefer to treat with the doses as high as 400 mg per day Even after an episode of angle closure, topiramate can be continued at a low dose of 12.5 mg per day and is reported to be less associated with recurrence. However, there have been reports of angle closure glaucoma with plasma topiramate levels less than therapeutic levels.[5] Our patient was on only 25 mg per day of the drug, which suggests that the reaction is not a dose dependent toxicity.

Macular striae were demonstrated on the OCT in our case. Fine folds at the level of the internal limiting membrane (ILM) were seen with resolution and restoration of normal visual acuity along with resolution of myopia and angle closure after discontinuation of topiramate [Figure 1b]. Although macular striae have been reported previously they have not been demonstrated by OCT[7,8] and were thought to be due to the vitreomacular traction.[7] Topiramate has effects on sodium and chloride movement that can interfere with ionic concentration in various tissues, including the crystalline lens. Elevation of the retina is secondary to fluid consistent with drug-induced altered membrane potential.[9] We hypothesize that the macular striae due to ILM folds may be due to the volume effect of the choroidal effusion. Spectral domain OCT with a better axial resolution (4.5 microns) is ideal for recording the fine striae. OCT due to its limited penetration into the choroid could not image the choroidal effusion. B scan, however, showed diffuse thickening of the choroid.

This case report illustrates angle closure, acute myopia, and retinal striae occurring even with low doses of topiramate. UBM evaluation of such a presentation is of immense value in ruling in this diagnosis.
